# Fatty acids linked to cardiovascular mortality are associated with risk factors

**DOI:** 10.3402/ijch.v74.28055

**Published:** 2015-08-12

**Authors:** Sven O. E. Ebbesson, Venkata S. Voruganti, Paul B. Higgins, Richard R. Fabsitz, Lars O. Ebbesson, Sandra Laston, William S. Harris, John Kennish, Benjamin D. Umans, Hong Wang, Richard B. Devereux, Peter M. Okin, Neil J. Weissman, Jean W. MacCluer, Jason G. Umans, Barbara V. Howard

**Affiliations:** 1 Department of Neurological Surgery, University of Virginia, Charlottesville, VA, USA; 2 Norton Sound Health Corporation, Nome, AK, USA; 3 Department of Nutrition and UNC Nutrition Research Institute, University of North Carolina at Chapel Hill, Kannapolis, NC, USA; 4 Kunming Biomed International, Yunnan, China; 5 National Heart, Lung, and Blood Institute, Bethesda, MD, USA; 6 Uni Targeting Research AS, Bergen, Norway; 7 Texas Biomedical Research Institute, San Antonio, TX, USA; 8 Department of Medicine, Sanford School of Medicine, University of South Dakota, Sioux Falls, SD, USA; 9 Health Diagnostic Laboratory, Inc., Richmond, VA, USA; 10 Department of Chemistry, University of Alaska Anchorage, Anchorage, AK, USA; 11 Department of Statistics, University of Oxford, Oxford, UK; 12 MedStar Health Research Institute, Hyattsville, MD, USA; 13 Weill Cornell Medical College, New York, NY, USA; 14 Georgetown-Howard Universities Center for Clinical and Translational Science, Washington, DC, USA

**Keywords:** Alaska Natives, cardiovascular risk factors, dietary fat consumption, fatty acids, fish oil consumption, Inuit, saturated fatty acids

## Abstract

**Background:**

Although saturated fatty acids (FAs) have been linked to cardiovascular mortality, it is not clear whether this outcome is attributable solely to their effects on low-density lipoprotein cholesterol (LDL-C) or whether other risk factors are also associated with FAs. The Western Alaskan Native population, with its rapidly changing lifestyles, shift in diet from unsaturated to saturated fatty acids and dramatic increase in cardiovascular disease (CVD), presents an opportunity to elucidate any associations between specific FAs and known CVD risk factors.

**Objective:**

We tested the hypothesis that the specific FAs previously identified as related to CVD mortality are also associated with individual CVD risk factors.

**Methods:**

In this community-based, cross-sectional study, relative proportions of FAs in plasma and red blood cell membranes were compared with CVD risk factors in a sample of 758 men and women aged ≥35 years. Linear regression analyses were used to analyze relations between specific FAs and CVD risk factors (LDL-C, high-density lipoprotein cholesterol, triglycerides, C-reactive protein, systolic blood pressure, diastolic blood pressure, heart rate, body mass index, fasting glucose and fasting insulin, 2-hour glucose and 2-hour insulin).

**Results:**

The specific saturated FAs previously identified as related to CVD mortality, the palmitic and myristic acids, were adversely associated with most CVD risk factors, whereas unsaturated linoleic acid (18:2n-6) and the marine n-3 FAs were not associated or were beneficially associated with CVD risk factors.

**Conclusions:**

The results suggest that CVD risk factors are more extensively affected by individual FAs than hitherto recognized, and that risk for CVD, MI and stroke can be reduced by reducing the intake of palmitate, myristic acid and simple carbohydrates and improved by greater intake of linoleic acid and marine n-3 FAs.

Although high saturated fat intake has been implicated in cardiovascular disease (CVD) and atherosclerosis for more than a century (1,2), specific dietary saturated fatty acids (FAs) and their related desaturases have only recently been shown to be associated with CVD mortality (3,4). Concurrently, evidence has emerged that unsaturated FAs (linoleic acid [LA] and long-chain omega-3 FAs) are associated with lower CVD mortality (3,5–7). The Uppsala Longitudinal Study of 2,000 Adult Men (ULSAM) found that serum cholesterol ester FA profiles were related to CV and total mortality over 30 years (3). The saturated FAs (myristic [14:0]) and palmitic [16:0] acids) and the unsaturated FAs (palmitoleic [16:1], γ-linolenic [18:3-6] and dihomo-γ-linolenic [20:3-6] FAs) (3) also were associated with CV mortality, whereas LA (18:2-6) concentration was negatively associated with 30-year CV and total mortality (3).

CVD is a multi-factorial disease, involving inflammation (8,9), hypertension (10), obesity (11,12), insulin resistance (13,14), diabetes (15,16), elevated heart rate (17–19), low high-density lipoprotein cholesterol (HDL-C) (20), high low-density lipoprotein cholesterol (LDL-C) (20) and/or high triglycerides (21).

Several randomized trials of the associations of saturated and unsaturated fatty acids and CVD have been conducted (22–27). Early studies substituting polyunsaturated fat for saturated fat showed reduction in CVD for high-risk participants. Later studies have focused on omega 3 fats; while these reports have not been consistent, the consensus is that omega 3 fats may lower the risk of sudden death in patients with CVD (5,28).

Investigators have suggested that LDL-C concentration is the main mediator between dietary saturated FAs and CVD (29–31), but other risk factors, such as insulin resistance, inflammation and endothelial function, are also affected by FAs (13,14,32). Therefore, the following questions arise: (a) which FAs are associated with specific CVD risk factors and (b) what is the most clinically relevant measure of FA status – its relative per cent composition in red blood cell (RBC) membranes or its concentrations in plasma (33).

The Genetics of Coronary Artery Disease in Alaska Natives (GOCADAN) study allows examination of these relationships in a population living in isolated villages along the northwest coast of Alaska. This population is currently undergoing a radical dietary change from traditional Native Alaskan foods, available primarily from the sea, to store-bought Western foods. This change involves a shift in dietary fat consumption from unsaturated FAs found in fish and sea mammals (34,35) to saturated FAs from farm animals, dairy products and manufactured fats (e.g. shortening). CVD incidence is changing concurrently. Whereas it was rare 40 years ago (<1/1,000) (36), CVD prevalence among Alaska Natives is now higher than that of the U.S. White population (37), and the prevalence and extent of carotid plaque in Western Alaskan Natives exceeds that of the U.S. White and African American populations (38) and is associated with blood concentrations of the saturated palmitic and myristic acids (39). Finally, stroke incidence in this population is 50% higher than that of the general U.S. population (40).

Measures of FAs used in previous studies range from dietary estimates, usually by food frequency questionnaire (FFQ) or multiple 24-hour recalls (3,41,42), to analyses of FA content in RBCs (35) or plasma (3,35) or through analyses of plasma lipid classes (either phospholipids or cholesterol esters) (3,43). Measurement errors and interpretation of all these measures vary. FFQs, however, may provide less reliable estimates of dietary FA intake than methods that measure FAs in blood (3,35,43) because they involve inaccuracies of memory, the inability of any questionnaire to completely reflect intake and because of incomplete nutritional databases, especially for traditional Alaska Native foods. The current study has, thus, been limited to analyses of FAs in plasma and RBCs (35), as these measures reflect the interplay between dietary and metabolic processes (44). Both of these measures have been validated as markers of intake of long-chain omega-3 FAs, *trans* FAs and, to some extent, LA (33–35).

Plasma concentrations of mono and saturated FAs do not entirely reflect diet as they can be modified by endogenous synthesis of carbohydrates (3), although this process is limited in those consuming high-fat Western diets (3). The total plasma FA biomarker used in the present study was used successfully as a biomarker previously in this population in a pilot intervention to reduce saturated fat consumption (34). This 4-year intervention resulted in a relative decrease of palmitate (16:0; p=0.02) concomitant with improved glucose tolerance (p=0.006), fasting glucose (p=0.0001), LDL cholesterol (p=0.0001) and diastolic blood pressure (p=0.0007), all without weight loss.

## Patients and methods

### Study population

A total of 1,214 predominantly Inupiat people >17 years of age from 9 villages in the Norton Sound Region of Alaska were examined between 2000 and 2004 for CVD and associated risk factors (45). In 7 of the 9 villages, an average of 82.6% of eligible residents participated (46). Screenings were terminated early in 2 additional villages, when the study reached its recruitment goal. From the sample of 1,214 participants, all participants ≥35 years of age (N=819; 361 men and 458 women) underwent assessment of total plasma and RBC FAs; those with previously diagnosed diabetes mellitus (DM) and on medication for DM (N=61) were excluded, yielding a study population of 758. The study was approved by the Research Ethics Review Board of the Norton Sound Health Corporation and by the institutional review boards of MedStar Health Research Institute and Texas Biomedical Research Institute. All participants provided informed consent.

### Study examination

The GOCADAN exam (45) consisted of a personal interview (including lifestyle assessment, medical history and medication use), physical examination (including ultrasound assessment of atherosclerosis in the carotid arteries), nutritional interview using a validated FFQ (42), anthropometric measurements (45), blood pressure (BP) measurements (45), heart rate measurement (18,19), electrocardiogram (ECG) (19,45), blood sampling, a Rose questionnaire (45), a Modifiable Activity Questionnaire (45), behavioural measures (45) and laboratory analyses (45). The methods for plasma and RBC FA composition have been described (19,35). Apolipoprotein E (ApoE) phenotype was determined in whole plasma by a modification of the Kamboh et al. method, in which 2 hours of isoelectric focusing are followed by immunoblotting to visualize the ApoE bands (45). Serum C-reactive protein (CRP) was assayed as described previously (8). Participants with CRP>10 (1.7% of sample) were excluded from the CRP analyses.

### Statistical analysis

Means, standard deviations and 95% confidence intervals were calculated to describe the population's characteristics. Concentrations of FAs in plasma and RBC membranes were compared with CVD risk factors in a sample of 758 men and women aged >34 years without diabetes, as defined by American Diabetes Association criteria (47). Prior to conducting analyses, distributional properties of all traits were evaluated. All values beyond 4 standard deviations were removed, and the remaining traits were transformed by inverse normalization prior to analysis to meet assumptions of normality.

While a correction for multiple testing was considered, it would have been inappropriate when the number of true positives (i.e. true biological associations that are also statistically significant) was likely to be a large percentage of the multiple tests. For the current analysis, a Bonferroni correction was not applied, because (a) RBC FA concentrations are less associated with some risk factors than those in plasma and (b) the Bonferroni adjustment is conservative and tends to produce false negatives. The conclusions expressed in this article are based on the plasma and RBC FA data without Bonferroni adjustment, in concurrence with other studies of individual variables (3,18,19,21,33,35). In addition, associations were first identified by individual testing of each FA and risk factor adjusted only for age. This approach provided additional confidence in the final results and conclusions.

### Statistical modelling

The CVD risk factors were adjusted for age, sex, age^2^, age×sex, age^2^×sex, current smoking (Y/N), cholesterol medication and “physical activity” metabolic equivalents (METs). The age-squared covariate was included to account for the effect of age, which may have a non-linear relationship with the lipids. BP was adjusted for BP medication, and LDL-C and HDL-C were adjusted for ApoE phenotype. CVD risk factors evaluated were heart rate, systolic BP (SBP), diastolic BP (DBP), mean arterial pressure, HDL-C, LDL-C, triglycerides, CRP, 2-hour glucose, 2-hour insulin, fasting glucose, fasting insulin and body mass index (BMI).

### Bivariate analyses

Bivariate analyses were conducted with Sequential Oligogenic Linkage Analysis Routines (SOLAR; Texas Biomedical Research Institute, San Antonio, TX) (48). Multiple linear regression analyses accounting for correlations among family members were used to analyze associations of CVD risk factors with specific FAs, after adjustment for relevant covariates.

Phenotypic correlations were computed between FAs and CVD risk factors (49).

## Results

Population characteristics of the study sample are shown (Table I). Those of the full GOCADAN sample have been described (19,35,38,45). Previous data have shown median calories consumed from fat in the 35–40% range, with about 25% of the population maintaining a subsistence diet with saturated fat <10% of energy intake (42). Smoking rates are high, medication usage is low and the women have higher BMIs than the men but similar waist circumferences. Women have higher fasting insulin, heart rate and HDL-C than the men. The relevant dietary components of this cohort have been described elsewhere (42). The 3 top sources of saturated FAs (myristic, palmitic and stearic acids) found in the Western Alaskan Native diet are from store-bought foods, mostly butter, vegetable shortening (Crisco^©^), evaporated milk, beef, cheese, creamer and chicken (42).

**Table I:** Population characteristics, Genetics of Coronary Artery Disease in Alaska Natives

	Women				Men			
		
	N	Mean	SD	Median	N	Mean	SD	Median
CVD risk factors								
Systolic BP (mmHg)	419	120.60	(16.8)	118	336	122.35	(14.2)	122
Diastolic BP (mmHg)	420	75.20	(9.2)	74	337	78.46	(9.58)	79
Mean arterial BP (mmHg)	419	105.47	(12.9)	104	336	106.94	(107.71)	107.3
BMI (kg/m^2^)	419	28.65	(6.2)	27.6	334	26.69	(5.1)	25.6
Waist (cm)	414	89.25	(14.2)	87.63	330	88.77	(12.0)	86.36
HDL-C (mg/dL)	419	66.94	(18.7)	65	337	57.39	(19.0)	54
LDL-C (mg/dL)	419	122.43	(35.1)	117	337	123.11	(36.6)	119
Triglyceride (mg/dL)	419	133.55	(77.8)	114	337	125.32	(70.8)	107
HbA1c (%)	421	5.51	(0.46)	5.41	337	5.53	(0.54)	5.5
Fasting glucose (mg/dL)	421	96.79	(17.8)	92	337	96.81	(25.1)	93
Fasting insulin (µU/mL)	420	10.86	(7.9)1	8.85	335	9.62	(9.2)	7.21
2-hour glucose (mg/dL)	282	107.54	(42.4)	102	237	93.35	(38.4)	88
2-hour insulin (µU/mL)	280	44.86	(41.9)	31.65	237	28.40	(37.4)	16.4
Heart rate (beats/minute)	383	73.75	(10.4)	72	324	71.19	(13.1)	70
C-reactive protein	420	0.426	(1.7)	0.12	337	0.338	(0.7)	0.11
Covariates								
METS	337	56.62	(58.1)	37.51	289	68.95	(66.1)	49.27
Age (year)	421	51.97	(12.2)		337	51.01	(11.7)	
Current smoking (%)	421	53			337	60		
Cholesterol medication (%)	421	9.5			337	7.4		
BP medication (%)	421	22			337	19		
ApoE (n)	421				337			
ApoE2 (%)	1				2			
ApoE3 (%)	73				66			
ApoE4 (%)	32				28			

Abbreviations: ApoE=apolipoprotein E; BP=blood pressure; BMI=body mass index; CRP=C-reactive protein; HDL-C=high-density lipoprotein cholesterol; LDL-C=low-density lipoprotein cholesterol; METS=metabolic equivalents.

The relative concentrations of some FAs differ significantly in plasma versus RBCs (Table II). Proportions of ω-3 FAs, 20:4-6 and 18:0, in RBC membrane phospholipids are much higher than those in plasma (50). On the contrary, proportions of 14:0, 16:0, 16:1-7, 18:1-9 and 18:2-6 are much higher in plasma than in RBCs.

**Table II:** Fatty acid proportions (% of total fatty acids) as measured in plasma (bold) and red blood cells (black), Genetics of Coronary Artery Disease in Alaska Natives

	Male (age in years)					Female (age in years)				
		
Fatty acid	35–44	45–54	55–64	65–74	p	35–44	45–54	55–64	65–74	p
Total omega 3	**4.86** 9.92	**5.34** 10.76	**5.94** 12.79	**5.96** 12.47	**0.1428** <0.0001	**5.29** 11.21	**5.57** 11.81	**5.77** 12.85	**7.14** 15.37	**0.0097** <0.0001
18:3-3	**0.56** 0.21	**0.55** 0.22	**0.57** 0.21	**0.61** 0.24	**0.8893** 0.8758	**0.6** 0.23	**0.57** 0.22	**0.56** 0.24	**0.64** 0.22	**0.4561** 0.6777
20:5-3	**1.13** 1.57	**1.3** 1.91	**1.44** 2.56	**1.88** 2.65	**0.0444** 0.0003	**1.14** 1.93	**1.36** 2.17	**1.35** 2.56	**1.78** 3.45	**<0.0001** <0.0001
22:5-3	**0.56** 2.33	**0.59** 2.44	**0.6** 2.69	**0.58** 2.61	**0.3165** 0.0018	**0.59** 2.51	**0.54** 2.48	**0.51** 2.58	**0.67** 2.91	**0.0527** 0.0042
22:6-3	**2.14** 5.81	**2.47** 6.18	**2.57** 7.34	**2.7** 6.97	**0.0948** <0.0001	**2.45** 6.53	**2.67** 6.94	**2.72** 7.48	**3.52** 8.78	**0.0020** <0.0001
C20-C22	**4.19** 9.71	**4.71** 10.53	**5.15** 12.58	**5.27** 12.23	**0.2125** <0.0001	**4.41** 10.97	**4.92** 11.59	**4.93** 12.61	**6.55** 15.14	**0.0001** <0.0001
Total omega 6	**35.06** 35.34	**29.35** 34.23	**27.81** 32.93	**32.32** 32.98	**0.3833** 0.0003	**30.17** 34.16	**31.27** 33.12	**35.34** 31.56	**29.27** 29.53	**0.7880** <0.0001
18:2-6	**28.64** 18.39	**28.04** 18.17	**26.82** 17.84	**25.48** 17.28	**0.0153** 0.2971	**27.57** 18.08	**26.78** 17.21	**24.76** 16.52	**23.84** 15.3	**<0.0001** <0.0001
20:4-6	**1** 13.06	**1.07** 12.59	**0.94** 11.96	**1.08** 12.37	**0.2567** 0.0013	**1** 12.5	**1.05** 12.35	**1.02** 11.95	**1.13** 11.49	**0.4566** 0.0053
Total MUFA	**22.95** 16.47	**22.78** 16.83	**24.28** 16.86	**23.21** 17.13	**0.8910** 0.0709	**22.34** 16.64	**22.79** 17.07	**23.81** 17.34	**23.38** 17.15	**0.5446** 0.0617
16:1-7	**2.11** 0.58	**2.29** 0.7	**2.3** 0.72	**2.54** 0.8	**0.0010** 0.0031	**2.57** 0.84	**2.67** 0.94	**2.76** 1.04	**2.83** 1.06	**0.0509** 0.0094
18:1-9	**18.96** 15.2	**19.29** 15.5	**19.62** 15.47	**20.1** 15.65	**0.3088** 0.2088	**18.82** 15.17	**18.9** 15.43	**18.85** 15.66	**18.81** 15.49	**0.9951** 0.1887
20:1-9	**0.13** 0.24	**0.18** 0.18	**0.17** 0.2	**0.19** 0.21	**0.3633** <0.0001	**0.14** 0.2	**0.13** 0.2	**0.13** 0.18	**0.11** 0.15	**0.6349** <0.0001
24:1-9	**0.37** 0.45	**0.3** 0.45	**0.4** 0.48	**0.2** 0.46	**0.8989** 0.7724	**0.41** 0.44	**0.38** 0.49	**0.52** 0.46	**0.42** 0.45	**0.5477** 0.1418
Total SFA	**31.9** 35.06	**31.14** 35.19	**31.15** 34.42	**31.74** 34.36	**0.3279** 0.3815	**32.24** 34.98	**32.51** 35.09	**33.57** 35.38	**33.34** 35.22	**0.0375** 0.3801
14:0	**0.89** 0.34	**0.88** 0.39	**0.79** 0.34	**0.86** 0.38	**0.4904** 0.1697	**0.98** 0.42	**0.97** 0.41	**1.05** 0.44	**1.11** 0.46	**0.1615** 0.2431
16:0	**23.81** 20.49	**23.09** 20.82	**23.15** 20.34	**23.81** 20.51	**0.2858** 0.2305	**24.14** 21	**24.29** 21.02	**25.15** 21.37	**24.87** 21.37	**0.0767** 0.0766
18:0	**7.19** 14.23	**7.17** 13.97	**7.21** 13.73	**7.07** 13.47	**0.9178** 0.1867	**7.12** 13.56	**7.24** 13.65	**7.38** 13.57	**7.35** 13.4	**0.4019** 0.942
Total trans FAs	**1.61** 2.24	**1.48** 2.08	**1.39** 2.09	**1.45** 2.17	**0.1042** 0.1144	**1.53** 2.14	**1.7** 2.04	**1.58** 2.03	**1.44** 1.92	**0.1539** 0.0869

Associations with risk factors also differ (Tables III and IV). The associations of RBC FAs differ from those of plasma FAs because (a) they reflect diet over 1–2 months instead of 1–2 days, (b) FAs in RBCs are bound to phospholipids only and (c) they reflect the tissue-selective incorporation of FAs into the RBC membranes. The total plasma FAs reflect those FAs bound to phospholipids and to cholesteryl esters.

**Table III:** Associations^a^ between CVD risk factors and total plasma fatty acids (µg/ml), Genetics of Coronary Artery Disease in Alaska Natives

0	14:0 Myristic	16:0 Palmitic	16:1-7 Palmitoleic	18:3-6 γ-Linolenic	20:3-6 Dihomo-γ-linolenic	18:0 Stearic	18:1 Oleic	18:2-6 Linoleic	20:4-6 Arachidonic	18:3-3 α-Linolenic	20:5-3 Eicosapentaenoic	22:6-3 Docosahexaenoic
Mortality	**+**	**+**	**+**	**+**	+	NS	**+**	**–**	NS	NS	NS	NS
Triglyceride	0.587 (5.2E^**−**29^)	0.440 (7.9E^**−**16^)	0.469 (1.8E^**−**23^)	0.250 (1.4E^**−**06^)	0.344 (3.3E^**−**13^)	0319 (4.7E^**−**09^)	0.377 (1.4E^**−**15^)	0.209 (8.9E^**−**06^)	0	0.421 (4.5E^**−**15^)	0.119 (0.047)	0
LDL-C	0.115 (0.03)	0.169 (0.002)	0.205 (0.000)	NS	0.113 (0.031)	0.235 (0.0000)	0.198 (0.0001)	0.249 (1.2E^**−**06^)	0.217 (0.0000)	0	0.254 (4.5E^**−**06^)	0.337 (8.9E^**−**10^)
HDL-C	**−0.263 (4.0E^−7^)**	NS	**−0 (0.028)**	NS	NS	NS	−0.137 (0.009)	NS	NS	**−0.146 (0.017)**	NS	NS
CRP	NS	NS	NS	NS	NS	NS	NS	NS	NS	NS	**−0.108 (0.026)**	NS
SBP	0.096 (0.031)	0.097 (0.029)	NS	0.110 (0.023)	0.137 (0.002)	NS	NS	NS	NS	NS	NS	NS
DBP	0.114 (0.013)	NS	NS	0.115 (0.021)	0.107 (0.021)	NS	0.091 (0.048)	NS	NS	NS	NS	NS
ABP	0.107 (0.018)	0.101 (0.024)	NS	0.118 (0.015)	0.143 (0.002)	NS	0.096 (0.031)	NS	NS	NS	NS	NS
HR	NS	NS	NS	NS	NS	NS	0	NS	**−0.168 (0.0002)**	NS	**−0.209 (0.0000)**	**−0.219 (0.0000)**
BMI	0.255 (1.2E^07^)	0.199 (0.0000)	0.172 (0.001)	0.174 (0.001)	0.197 (0.0001)	0.190 (0.0001)	0.161 (0.001)	NS	0.123 (0.012)	0.175 (0.003)	NS	NS
Waist	0.280 (1.5E^**−**08^)	0.209 (0.0000)	0.187 (0.0000)	0.204 (0.0002)	0.193 (0.0001)	0.189 (0.0001)	0.180 (0.0002)	NS	0.137 (0.006)	0.166 (0.005)	NS	NS
% fat	0.268 (1.2E^**−**07^)	0.183 (0.0004)	NS	0.207 (0.0001)	0.198 (0.0001)	0.159 (0.0018)	NS	NS	NS	NS	NS	NS
2-hour glucose	0.175 (0.0021)	0.166 (0.004)	0.200 (0.0005)	NS	0.181 (0.001)	NS	0.159 (0.005)	NS	NS	NS	NS	NS
2-hour insulin	0.160 (0.007)	0.138 (0.023)	(0.0001)	0.146 (0.026)	0.165 (0.005)	NS	0.119 (0.046)	NS	NS	0.150 (0.018)	NS	NS
Fasting glucose	0.100 (0.035)	NS	NS	NS	NS	NS	NS	NS	NS	NS	NS	NS
Fasting insulin	0.282 (2.88E^09^)	0.195 (0.0000)	NS	0.137 (0.0065)	0.23 (0.0000)	0.186 (0.0001)	NS	NS	NS	NS	NS	NS
HbA1c	0.141 (0.003)	NS	NS	0.191 (0.003)	NS	NS	NS	NS	NS	NS	NS	NS

a Results are presented as phenotypic correlation estimate (p-value). Negative associations are shown in bold. All phenotypes are adjusted for age, age^2^, sex, age×sex, age^2^×sex, BMI and physical activity. BMI was not used as a covariate for the BMI analysis. Additional covariates were used as follows: lipid medications for lipids, antihypertensive medication for HR and SBP and smoking for CRP.

Mortality data are from Warensjo et al. (3). BMI=body mass index; BP=blood pressure; CRP=C-reactive protein; DBP=diastolic blood pressure; HDL-C=high-density lipoprotein cholesterol; HR=heart rate; LDL-C=low-density lipoprotein cholesterol; MAP=mean arterial pressure; SBP=systolic blood pressure; NS=not significant.

**Table IV:** Associations between CVD risk factors and% of total fatty acids in RBCs^a^, Genetics of Coronary Artery Disease in Alaska Natives

	14:0 Myristic	16:0 Palmitic	16:1-7 Palmitoleic	18:3-6 γ-Linolenic	20:3-6 Dihomo-γ-linolenic	18:0 Stearic	18:1 Oleic	18:2-6 Linoleic	20:4-6 Arachidonic	18:3-3 α-Linolenic	20:5-3 Eicosapentaenoic	22:6-3 Docosahexaenoic
Mortality	**+**	**+**	**+**	**+**	**+**	NS	**+**	**–**	NS	NS	NS	NS
Triglyceride	0.31 (0.0000)	0.33 (0.0000)	0.26 (0.0000)	0.28 (0.000)	0.211 (0.0000)	NS	0.231 (0.0000)	NS	NS	0.17 (0.003)	**−0.315 (0.0000)**	**−0.20 (0.0005)**
LDL-C	NS	NS	NS	**−0.146 (0.008)**	**−0.228 (0.0000)**	NS	NS	NS	**−0.177 (0.001)**	NS	0.20 (0.0002)	0.173 (0.002)
HDL-C	NS	NS	NS	NS	NS	NS	NS	0.122 (0.026)	NS	NS	NS	NS
CRP	NS	0.091 (0.045)	NS	NS	0.158 (0.0004)	NS	NS	NS	0.100 (0.037)	NS	**−0.106 (0.018)**	**−0.113 (0.011)**
SBP	NS	0.146 (0.006)	NS	NS	0.175 (0.0000)	NS	0.109 (0.042)	NS	NS	NS	NS	NS
DBP	NS	0.126 (0.0024)	NS	NS	0.195 (0.0000)	0.144 (0.010)	NS	NS	NS	NS	**−0.152 (0.007)**	**−0.117 (0.039)**
MAP	NS	0.148 (0.006)	NS	NS	0.191 (0.0000)	NS	NS	NS	NS	NS	NS	NS
HR	NS	0.178 (0.0001)	0.137 (0.002)	NS	0.244 (0.0000)	NS	NS	NS	NS	NS	**−0.116 (0.010)**	**−0.113 (0.012)**
BMI	0.128 (0.009)	0.146 (0.003)	NS	0.103 (0.039)	0.091 (0.066)	NS	NS	**−0.127 (0.009)**	NS	NS	NS	NS
Waist	0.161 (0.001)	0.187 (0.0002)	0.124 (0.014)	0.124 (0.015)	0.100 (0.048)	NS	NS	**−0.135 (0.007)**	NS	NS	NS	NS
2-hour glucose	NS	0.216 (0.0001)	0.215 (0.0001)	NS	0.118 (0.033)	NS	NS	**−0.157 (0.005)**	NS	NS	NS	NS
2-hour insulin	NS	0.115 (0.048)	NS	0.149 (0.008)	0.168 (0.004)	NS	NS	NS	NS	NS	**−0.150 (0.009)**	NS
Fasting glucose	NS	NS	NS	NS	NS	NS	NS	**−0.103 (0.032)**	NS	NS	NS	NS
Fasting insulin	0.171 (0.0003)	NS	NS	0.124 (0.010)	NS	NS	NS	NS	**−**0.106 (0.028)	NS	NS	NS

a Results are presented as phenotypic correlation estimate (p-value). Negative associations are shown in bold. All phenotypes are adjusted for age, age^2^, sex, age×sex, age^2^×sex, BMI and physical activity. BMI was not used as a covariate for the BMI analysis. Additional covariates were used as follows: lipid medications for lipids, antihypertensive medication for HR and SBP and smoking for CRP.

Mortality data are from Warensjo et al. (3). ABP=average blood pressure; BMI=body mass index; CRP=C-reactive protein; DBP=diastolic blood pressure; HDL-C=high-density lipoprotein cholesterol; HR=heart rate; LDL-C=low-density lipoprotein cholesterol; SBP=systolic blood pressure; NS=not significant.

The plasma FA profiles related to risk factors in the current study (Table III) are almost identical to those found related to CV mortality in the ULSAM study (3). In the present study, total plasma FA concentrations of saturated FAs (14:0 and 16:0) and those associated with high saturated FA consumption (16:1, 18:3-6, and 20:3-6) were in general positively associated with CVD risk factors (triglycerides, LDL-C, HDL-C [negatively], CRP, SBP and DBP, heart rate, BMI, and fasting glucose and fasting insulin, 2-hour glucose and 2-hour insulin), while the polyunsaturated FAs (PUFAs), LA (18:2-6), eicosapentaenoic acid (EPA, 20:5-3) and docosahexaenoic acid (DHA, 22:6-3) were primarily either inversely or not associated with CVD risk factors (Table III).

## Discussion

The results support the hypothesis that specific FAs previously identified as related to CV mortality (3) are associated with specific CVD risk factors. The ULSAM investigators found no association between CVD and marine FAs (20:5-3 and 22:6-3), perhaps because these FAs are not well represented in the plasma cholesterol esters they studied (3). For similar reasons, estimates of associations between lipoprotein cholesterol and RBC FAs reported in the current study are of questionable significance because the FAs related to lipoprotein cholesterols are likely bound to plasma lipoproteins (Table III) but not to the RBC FAs in membranes (Table IV) (51–53).

Our results explain and are in agreement with other clinical data. The clinical repercussions of overconsumption of certain saturated FAs are clear. Research has shown that serum FA profiles with high palmitic acid, palmitoleic acid (16:1) and dihomo-γ-linolenic acid (20:3-6), and low proportions of LA (18:2-6) predict myocardial infarction (54,55), stroke (56), left ventricular hypertrophy (57) and metabolic syndrome (21,58,59). It is also known that high saturated FA consumption increases insulin resistance (35,58), glucose intolerance (35,58), metabolic syndrome (58,60) and low-grade inflammation (9,14).

The sources of the clinically relevant FAs in this study population are summarized as follows:

*Myristic acid* (14:0) comes mostly from store-bought foods (butter, cream, whole milk and tropical oils).

*Palmitic acid* (16:0) comes mainly from store-bought items (42), but it is also manufactured in the liver (3). This FA has been associated with impaired glucose tolerance and insulin resistance (14,36,57); development of type 2 DM (34,35); and increased BP (35,58), BMI (61), carotid plaque and plaque score (62). The broad effects of this FA on CVD risk factors correspond with data from a pilot study to reduce saturated FA consumption in another cohort of Western Alaska Natives, which resulted in reductions in plasma concentrations of palmitate (16:0), stearate (18:0), LDL-C, fasting glucose, DBP and improved glucose tolerance (35).

*Palmitoleic acid* (16:1) is found in macadamia oil and marine mammals (including seal oil), but it is also a product of endogenous lipogenesis. It is biosynthesized from palmitate by D9D, which is upregulated by saturated fat consumption (3) and behaves like a saturated FA, increasing insulin resistance (14), heart rate (19) and cholesterol (3). In fact, 16:1-7 raises LDL-C more than palmitic acid (16:0) (63). Palmitoleic acid (16:1-7) and its associated D9D activity predicted mortality most strongly in the Swedish cohort, where it was associated with abdominal obesity and triglyceridemia (3). Because D9D catalyses the synthesis of 16:1-7 and 18:1-9 and is upregulated by saturated fat (3), the high intake of saturated fat results in increased lipogenesis and plays a key role in obesity-related diseases (64–66). In the current study, LDL-C was not associated with either RBC phospholipid-bound FAs 16:0 or 16:1, possibly because the phospholipid fractions of the FAs in RBC membranes have little to do with cholesterol metabolism.

*Stearic acid* (18:0) was not associated with CVD mortality in the Swedish study (3) and was only associated with DBP in the RBC samples in the current study. The relative proportion of 14:0 and 16:0 FAs is higher than 18:0 in meat from corn-fed cattle kept in feedlots than from grass-fed cattle (67), suggesting that the fat in store-bought meat available in village stores has a high proportion of the detrimental FAs 14:0 and 16:0 (42). In the current study, as in other studies, 18:0 appears mainly related to risk factors for obesity and type 2 DM (15).

*Gamma-linolenic acid* (18:3-6) is mainly a metabolite of 18:2-6 and is the precursor to *dihomo-γ-linoleic acid* (DHLA; 20:3-6), which is a precursor of arachidonic acid.

*Oleic acid* (18:1-9) is derived from olive oil, canola, butter, milk, eggs, seal oil and muktuk and can be synthesized de novo from stearic acid by the action of D9D. This FA is generally considered protective or neutral as related to CVD risk (68–70), although some researchers have reported increased CVD risk with higher intake (28). The latter association may be related to the mutual dietary sources of oleic acid and saturated FAs in dairy products, meat and poultry.

Concentration of *linoleic acid* (18:2-6) in serum is known to reflect dietary intake and is negatively associated with CVD mortality (3) and with a few CVD risk factors (Table IV). The positive association between LA and LDL-C in plasma samples of the current study cannot be explained. LA is known to lower LDL-C (29,30) and decrease risk of myocardial infarction (54). Such protective effects have been noted in clinical and prospective studies (3), and it has been learned in the past few years that FA profiles with low proportions of LA (18:2-6) predict myocardial infarction (4,55), stroke (56), left ventricular hypertrophy (57), insulin sensitivity (14), glucose intolerance (61) and metabolic syndrome (58,60). The conclusion must be that LA is protective (i.e. LA reduces some risk factors, thereby reducing risk for CVD and mortality).

Relative concentrations (%) of *alpha-linolenic acid* (ALA, 18:3-3) in serum was only marginally (p=0.06) associated with CV mortality (3) and in this study was associated with increased triglycerides, BMI and 2-hour insulin scores, suggesting that this FA may have some detrimental effects.

*Marine omega-3 FAs* (EPA, 20:5-3; and DHA, 22:6-3) in RBCs were negatively associated with triglycerides, CRP, DBP, HR and 2-hour insulin, but positively associated with triglycerides and LDL-C in plasma FAs. The ULSAM study found no association between EPA/DHA and CVD mortality, but that could have resulted from looking only at FAs in serum cholesterol esters, which lack high concentrations of these FAs (3). These FAs, in the Alaska Native population, are derived mainly from salmon, sardines, seal oil and whale blubber and are considered cardioprotective, as numerous studies have shown an inverse association with sudden cardiac arrest (28). The conclusion from studies in Greenland, that these FAs prevent atherosclerosis, has recently been cast into doubt as no association has been found with the presence of plaque in Alaska Natives (39). IMT, however, was inversely associated with EPA and DHA (39). The beneficial effects are now believed to be related to reducing arrhythmia (5,18,19,28), which is associated with heart rate, which in turn is negatively associated with RBC and plasma concentrations of EPA and DHA (19).

### Differences in FA biomarkers

The results from the 2 FA measures (plasma and RBCs) in the current study and from serum cholesterol ester measures in the ULSAM study (3) are similar in their identification of the FAs that are either detrimental or protective for CV health. Differences between the FA measures, however, are reflected in the associations of FAs with CV risk factors. The current study shows that the 2 measurement methods complement each other. The FA contents of RBCs have poor associations with cholesterol compared with the well-established associations observed with dietary and total serum FA measures (3,32). Plasma measures of saturated FAs were associated with plasma LDL-C concentrations in this cohort, possibly because FAs related to cholesterol are mainly bound to lipoproteins in the plasma. On the contrary, the FAs in serum cholesteryl esters are thought to contain insufficient amounts of marine ω-3 FA to provide reliable associations (3).

## Conclusions

High blood concentrations of saturated fats (14:0 and 16:0) are detrimental to cardiovascular health and the biological systems related to it, while some unsaturated FAs (18:2-6, 20:5-3, 22:6-3) are protective.

This study has many strengths. It is the largest population-based survey of CVD and its risk factors in Western Alaska Natives. All methods were carefully standardized and outcomes were ascertained according to American Heart Association/National Heart, Lung, and Blood Institute standard procedures (71). Weaknesses are due to the observational nature of the study, which precludes conclusions of cause and effect. In addition, there may be several dietary and behavioural residual confounders that have not been measured.

The associations between specific FAs and CVD risk factors observed here are consistent with the known relations between FAs and CVD (3). Although these FA biomarkers do not precisely reflect diet, our findings suggest that reducing plasma concentrations of detrimental saturated FAs may in turn improve overall CVD risk status. Reducing palmitate and simple carbohydrate consumption and increasing fish oil consumption could thus lead to a decrease in CVD, MI and stroke. The results, therefore, demand a well-controlled intervention study to reduce plasma concentrations of FAs linked to CVD mortality and CVD risk factors as identified in this article.
